# *In Vitro* Evaluation of *Candida albicans* Adhesion and Related Surface Properties of CAD/CAM Denture Base Resins

**DOI:** 10.1055/s-0043-1774319

**Published:** 2023-12-12

**Authors:** Shaimaa M. Fouda, Mohammed M. Gad, Reem Abualsaud, Passent Ellakany, Hamad S. AlRumaih, Faraz A. Farooqi, Asif Matin, Doaa M. Al-Eraky, Faisal D. Al-Qarni, Fahad A. Al-Harbi

**Affiliations:** 1 Department of Substitutive Dental Sciences, College of Dentistry, Imam Abdulrahman Bin Faisal University, Dammam, Saudi Arabia; 2 Department of Dental Education, College of Dentistry, Imam Abdulrahman Bin Faisal University, Dammam, Saudi Arabia; 3 IRC Membranes and Water Security, King Fahd University of Petroleum and Minerals, Dhahran, Saudi Arabia; 4 Department of Biomedical Sciences, College of Medicine, Gulf Medical University, Ajman, United Arab Emirates

**Keywords:** CAD/CAM, 3D printing, denture base resin, surface properties, *Candida albicans*, denture stomatitis

## Abstract

**Objective** The aim of this study was to evaluate the surface roughness, contact angle, and adhesion of *Candida albicans* to computer-aided designing/computer-aided manufacturing (CAD/CAM) and heat-polymerized (HP) denture base materials.

**Materials and Methods** Specimens were allocated to six groups based on the composition of studied denture base materials, HP acrylic resin, milled resins (AvaDent and IvoCad), and 3D-printed resins (ASIGA, FormLabs, and NextDent). Ten specimens per group were used for each test ( *n* = 10/test). Surface roughness and contact angles were analyzed using profilometer and goniometer, respectively. Adhesion of *C. albicans* was counted using colony-forming unit (CFU/mL). Means and standard deviations were calculated, and then one-way analysis of variance (ANOVA), followed by Tukey's post hoc test. Correlation of *Candida* adhesion and surface parameters was determined by using Pearson's correlation analysis.

**Results** No statistically significant difference was noted in surface roughness between HP, milled, and 3D-printed denture base resins except NextDent, which showed significantly higher roughness in comparison to all other resins ( *p* = 0.001). In terms of contact angle, milled resins had the lowest value, followed by HP, ASIGA, and FormLabs, whereas NextDent showed the highest contact angle ( *p* = 0.001). *C. albicans* adhesion showed no significant difference between all denture base resins. A positive and significant correlation was found between *C. albicans* adhesion and contact angle ( *p* = 0.003), while no correlation was reported between *C. albicans* adhesion and surface roughness ( *p* = 0.523).

**Conclusion** Adhesion of *C. albicans* was similar in all tested specimens. Surface roughness showed no significant difference between all groups except NextDent, which had the highest value. Milled denture base resins had the lowest contact angle among all groups.

## Introduction

Polymethyl methacrylate (PMMA) resin was proven satisfactory for the fabrication of removable dentures due to its mechanical, optical, aesthetic, and biological characteristics. 1 2 However, low surface hardness, porosities, surface roughness, and contact angles could enhance microbial adhesion. 2 Thus, epidemiological studies report a 70% prevalence of denture-associated stomatitis in patients wearing removable prosthesis, with *Candida albicans* being the main pathogen. 3

*Candida* adhesion to acrylic surface has been correlated with the surface properties (roughness and hydrophobicity) of the material, the *Candida* species, and the surrounding environment. 4 5 The surface roughness of acrylic resin is dependent on many factors including the material's structure, manufacturing process such as polymerization, and polishing procedures. 6 Roughness and grooves on the resin specimen provide more hideout places for microorganisms away from the normal cleaning process. 4

Additionally, the microbial adhesion to a material is correlated to its surface hydrophobicity and free energy. 7 The hydrophobic *Candida* easily adheres to hydrophobic resin surface. Therefore, increasing the hydrophilicity and reducing contact angle could lower *Candida* adhesion. 4 Murat et al 8 described a significant positive association between surface roughness and *Candida* adhesion with no correlation between hydrophobicity and *Candida* adhesion. On the contrary, da Silva et al 1 stated that as the hydrophilicity of a denture base material is changed, microbial colonization is altered. In addition to the impact of surface properties on *Candida* adhesion, the residual monomer that is released from denture base material over time may lead to porosity formation and enhance the adhesion of *Candida* and biofilm formation thereafter. 9

As surface properties play an essential role in *Candida* adhesion, altering these characteristics may make the dentures less prone to the adhesion. 2 These alterations may comprise surface coating of the resin, chemical composition modification, or the addition of fillers. All of these treatment modalities proved effective in reducing *Candida* adhesion when contact angle and surface roughness were reduced. 4 A simpler approach may involve the use of computer-aided designing/computer-aided manufacturing (CAD/CAM) PMMA as a substitute to heat polymerization. The manufacturing technique of CAD/CAM milled PMMA creates a highly cross-linked structure that is less porous with minimal residual monomer. 9 Additionally, milled dentures have a better fit, which reduces dead spaces under the denture that acts as a *Candida* reservoir. 8 10

On the other hand, dentures can be made utilizing the 3D-printing technology, where they are virtually designed using CAD software and then 3D printed using the desired resin material. 11 Nevertheless, this technology is relatively new to the removable prosthesis field and has not been extensively investigated. Few studies evaluated the surface characteristics of various kinds of denture base resins and reported conflicting results. Di Fiore et al 12 reported insignificant differences between the surface roughness of heat-polymerized (HP), milled, and 3D-printed denture base resins after regular polishing procedures, while Gad et al 13 reported lower surface roughness of 3D-printed PMMA compared with HP resin. Other studies reported significant differences in roughness and contact angle between various types of 3D-printed resins and in comparison to milled or HP resins. 14 15

The surface roughness and wettability might vary according to the brands of CAD/CAM PMMA used. 16 Accordingly, the present study assessed the surface roughness, contact angle, and *C. albicans* adhesion among different brands of CAD/CAM denture base resins manufactured by different CAM technologies (milling and 3D printing) in relation to conventional HP denture base resins. The study's null hypothesis stated that there will be no difference in surface roughness, contact angles, and *C. albicans* adhesion between CAD/CAM and conventional HP denture base resins.

## Materials and Methods

### Sample Size Calculation and Test Groups

The sample size for this study was determined through power analysis. For this purpose, the formula was adopted from the World Health Organization (WHO), keeping 0.05 as the level of significance, power at 80%, and marginal error set at 5%, which demonstrated the need for 10 specimens for each group to estimate the presumed effect size. The total number of required specimens was 180 divided as follow; 60 specimens per tested property with 10 specimens of each material. Rectangular acrylic specimens with dimension of 10 × 12 × 2.5 mm were prepared from different resins: HP acrylic resin, prepolymerized acrylic disks for milling, AvaDent and IvoCad and 3D-printed resins, ASIGA, FormLabs, and Denture 3D+ (see Table 1 for details).

**Table 1:** Materials used in the present study

Group	Material/equipment
Heat-polymerized acrylic resin	Major.Base.20, Major Prodotti Dentari, Moncalieri, Italy
AvaDent	AvaDent denture base puck (AvaDent, Global Dental Science Europe, Tilburg, the Netherlands)
IvoCad	IvoBase CAD (Ivoclar Vivadent, Schaan, Liechtenstein)
ASIGA	(Resin) ASIGA DentaBASE (Asiga pty Ltd, Alexandria, NSW, Australia) (Printer) ASIGA MAX Printer (DLP)
FormLabs	(Resin) FormLabs Denture Base LP (FormLabs, Somerville, MA, United States) (Printer) Form 2 Printer (SLA)
NextDent	(Resin) Denture 3D+ (NextDent B.V., Soesterberg, the Netherlands) (Printer) NextDent 5100 3D Printer (SLA)

### Fabrication of HP, Milled, and 3D-Printed Specimens

HP (control) acrylic resin specimens were fabricated by the use of conventional water bath method as mentioned in an earlier study. 17 Investing of wax specimens in dental stone was done, followed by wax elimination. Packing of acrylic resin mix was done at the dough phase following the application of separating medium on stone surfaces. After that, processing of acrylic resin was achieved in water bath polymerization unit (KaVo Elektrotechnisches Werk GmbH, Leutkirch, Germany) at 73°C for 90 minutes, then at 100°C for an additional 30 minutes. Finishing of specimens was accomplished by the use of tungsten carbide bur (HM 79GX-040 HP; Meisinger, Centennial, CO, United States) to remove excess resin.

For both milled groups (AvaDent and IvoCad), prepolymerized PMMA pucks were cut to the required dimensions, where each disk was positioned and fixed in precision cutting machine (IsoMet 5000 Linear Precision Saw, Buehler, Lake Bluff, IL, United States) and sliced using diamond disk under constant water coolant. 18

For the printed specimens, the stereolithography (STL) file of the design was created using an open software (123D design, Autodesk, version 2.2.14, San Francisco, California, United States) and sent to each material's corresponding printer ( Table 1 ). For ASIGA and NextDent resins, the resin containers were shaken for 30 minutes and then poured into the resin tank, while for FormLabs, the resin tank was mounted on the printer directly. The printing orientation of all specimens was set at 90 degrees to the platform and 50-µm layer thickness. Following the printing procedure, the specimens were immersed in 99.9% isopropyl alcohol (IPA) to remove uncured resin. To complete the polymerization of printed specimens, additional postcuring cycle was done according to manufacturer's recommendations. Specimens were placed in the glycerin path within the postcuring machine.

All specimens (HP, milled, and 3D printed) were polished using 1,200-grit sandpaper disks (MicroCut PSA; Buehler) by the use of a polishing machine (MetaServ 250 Grinder Polisher, Buehler) in wet settings to ensure standardized polishing methods. A single investigator performed the polishing procedure of all the specimens and reassessed the specimens' dimensions to 0.01-µm accuracy using a digital caliber. Specimens with acceptable dimensions were incubated in distilled water at 37°C for 48 hours before assessing the desired properties.

### Measurement of Surface Roughness and Contact Angle

A noncontact optical interferometric profilometer (Contour GT; Bruker Nano GmbH, Berlin, Germany) was utilized in measuring the surface roughness ( *R _a_* ) of each specimen at five distant areas and 0.01-mm resolution. The average *R _a_* value per specimen was then calculated. 19

An automated goniometer (DM-501; Kyowa Interface Science Co., Niiza-City, Saitama, Japan) measured the contact angle (degrees) at four areas on each specimen followed by mean value calculation per specimen. The sessile drop technique was followed using an autopipette to dispense (2-μL) droplets of distilled water on the specimen's dry surface. The images were interpreted using the FAMAS software (Kyowa Interface Science Co.). 20

### Microbiological Analysis of the Biofilm

Frozen culture of *C. albicans* reference strain (ATCC 10231) was inoculated onto Sabouraud dextrose agar (SDA; MOLEQULE-ON, New Lynn, Auckland, New Zealand) for 48 hours at 37°C. Isolated colonies were added to Sabouraud dextrose broth (SDB; MOLEQULE-ON) for overnight incubation at 37°C and then the broth was diluted and adjusted to approximately 00.5 McFarland (1 × 10 ^7^ CFU/mL; DensiCHEK TM Plus, Durham, NC, United States).

The biofilm formation was evaluated using the protocol of colony-forming unit (CFU) assay according to Gulati et al 21 with a slight modification. Briefly, after sterilization of specimens with 70% IPA, each specimen was placed in 12-well cell culture plates (Nunclon Delta Surface, Thermo Fisher Scientific, Roskilde, Denmark), and a volume of 1,000 µL of the adjusted yeast suspension was added to each well and incubated at 37°C for 48 hours. To remove nonadherent cells, the specimens were washed three times with phosphate buffer saline (PBS), then scraped and vortex for 2 minutes at 3,000 rpm to dislodge the adherent cells from the specimens. 22 23 24 To enumerate CFU count, 10-fold dilution in PBS was performed, before a volume of 100 µL was directly platted onto SDA plates and incubated at 30°C for 48 hours. The experiment was performed blindly in triplicates with positive and negative controls to ensure reproducibility. 25 26 27

### Statistical Analysis

The normality of the data was evaluated using the Shapiro–Wilk test and *p* -values greater than 0.05 indicated that the data were normally distributed. Comparison of means between the groups (HP, AvaDent, IvoCad, ASIGA, FormLabs, and NextDent) for each tested property was done using one-way analysis of variance (ANOVA). Significant ANOVA results necessitated the use of Tukey's post hoc test to identify the pairwise differences. Pearson's correlation analysis was used to correlate *C. albicans* adhesion and related surface parameters. Statistical package for social science (SPSS, IBM, New York, United States) version 24 was used for statistical analysis and *p* -values ≤0.05 were considered statistically significant.

## Results

Table 2 presents the means, standard deviations, and significance of surface roughness ( *R _a_* , µm) between tested materials. ANOVA results for *R _a_* exhibited a significant difference between the groups ( *p* = 0.001). For pairwise comparisons, no significant differences in surface roughness were found between any of the groups except with NextDent, which showed the highest *R _a_* value (1.68 ± 0.22 µm) among the groups ( *p* < 0.05), whereas ASIGA showed the lowest *R _a_* value (0.92 ± 0.23 µm).

**Table 2:** Mean, SD, and significances between groups for all tested properties

	HP	AvaDent	IvoCad	ASIGA	FormLabs	NextDent	*p* -value
	Mean ± SD	Mean ± SD	Mean ± SD	Mean ± SD	Mean ± SD	Mean ± SD	
* R _a_* (µm)	1.09 ± 0.16 a	1.28 ± 0.41 a	1.15 ± 0.12 a	0.92 ± 0.23 a	1.23 ± 0.33 a	1.68 ± 0.22	*F* = 9.931 *p* = 0.001 b
Contact angle (degrees)	79.44 ± 3.84 a	70.01 ± 2.61 c	72.4 ± 3.74 c	81.63 ± 3.13 a	80.62 ± 8.35 a	89.91 ± 3.61	*F* = 23.709 *p* = 0.001 b
*Candida* count (log _10_ CFU/mL) d	4.22 ± 0.166	4.14 ± 0.089	4.11 ± 0.101	4.21 ± 0.118	4.21 ± 0.066	4.27 ± 0.203	*F* = 1.897 *p* = 0.110

Abbreviations: CFU, colony-forming unit; HP, heat-polymerized; SD, standard deviation.

a No statistical difference between the groups.

b Statistically significant at *p* = 0.05.

c No statistical difference between the groups.

d ANOVA (analysis of variance) results were not statistically significant; therefore, post hoc was not performed.

**Fig. 1:**
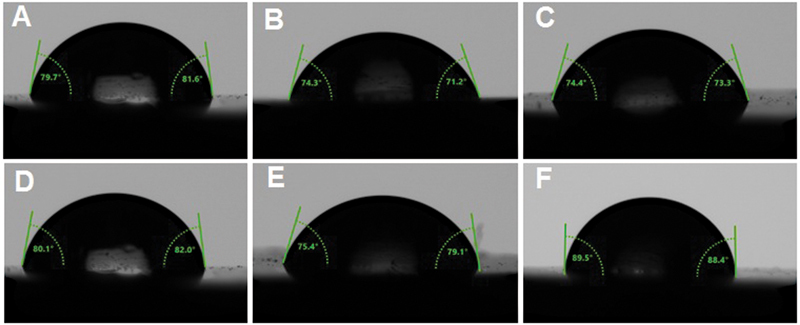
Representative images of contact angle of all tested resins. ( **A** ) heat-polymerized (HP), ( **B** ) AvaDent, **(C** ) IvoCad, ( **D** ) ASIGA, ( **E** ) FormLabs, and ( **F** ) NextDent.

For contact angle, the values are summarized in Table 2 , and representative images of contact angles are shown in Fig. 1 . The ANOVA results showed significant differences between the materials ( *p* = 0.001). For intergroup comparisons, NextDent significantly showed the highest contact angle (89.91 ± 3.61 degrees; *p* = 0.001) among the groups. Compared with the control material (HP), the milled groups (AvaDent/IvoCad) showed significantly lower contact angles ( *p* = 0.001 and 0.015, respectively), while ASIGA and FormLabs showed no significant differences ( *p* = 0.895 and 0.992, respectively).

**Fig. 2:**
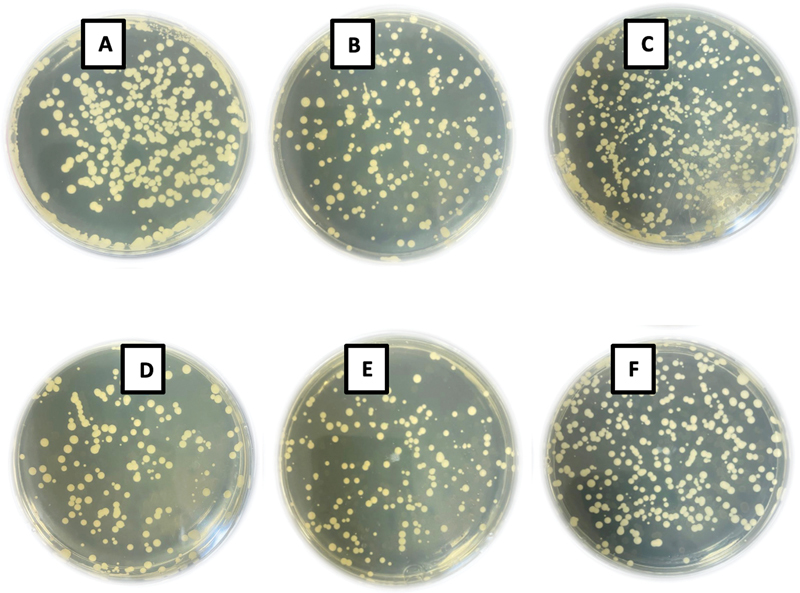
The colony forming unit assay of *Candida albicans* biofilm recovered from each tested group: ( **A** ) heat-polymerized (HP), ( **B** ) AvaDent, ( **C** ) IvoCad, ( **D** ) ASIGA, ( **E** ) FormLabs, and ( **F** ) NextDent.

The *C. albicans* colony counts per material are presented in Table 2 and Fig. 2 . The overall results showed that there was no significant difference in *C. albicans* count between all tested materials ( *p* = 0.074). The highest CFU (log _10_ CFU/mL) count of *C. albicans* adhered to the NextDent-printed specimens (4.27 ± 0.203), while the lowest count was recorded with IvoCad (4.11 ± 0.101).

Table 3 displays the level of association between *C. albicans* count and tested surface parameters that were analyzed using Pearson's correlation. The analysis showed that *C. albicans* count was not associated with surface roughness, but was significantly associated with contact angle.

**Table 3:** Pearson's correlation among *Candida* adhesion and surface parameters

	*Candida* count	* R _a_*	Contact angle
*Candida* count	1	*r* = 0.492 *p* = 0.149	*r* = 0.825 *p* = 0.003 a
* R _a_*		1	*r* = 0.230 *p* = 0.523
Contact angle			1

a Significant at *p* = 0.05.

## Discussion

The present study tested the surface roughness, contact angle, and *C. albicans* adhesion of HP denture base acrylic resin compared with the CAD/CAM counterparts fabricated by milling or 3D-printing technologies. The study hypothesis was partly rejected since the contact angle and surface roughness showed variation between the tested denture base resins, while *C. albicans* adhesion showed no significant difference.

The results demonstrated no significant differences between the surface roughness of HP, milled, and 3D-printed resins, except NextDent specimens, which showed the highest value among all tested groups. Similarly, Di Fiore et al 12 found that HP and CAD/CAM denture base resins (milled and 3D printed) had similar surface roughness after polishing, while the milled resin showed lower surface roughness before polishing. Also, Al-Dwairi et al 15 mentioned that 3D-printed resins showed various surface properties between different brands where ASIGA exhibited the lowest surface roughness among the studied 3D-printed resins, which is in agreement with our results. In contrast, previous studies reported lower surface roughness of milled and 3D-printed resins, in comparison to HP. 4 8 16 18 Also, Helal et al 14 and Alharethi 28 found lower surface roughness of milled resin compared with printed resins. Variation in surface roughness results might be related to differences between the tested materials, tested manufacturing techniques, or the polishing methods used in these studies. Also, some studies did not compare between milled and 3D-printed resins, 15 16 or included only one type (brand) of denture base resin for each fabrication technique (milling or 3D printing). 12 Murat et al 8 subjected the specimens to thermal cycling before testing the surface properties, which could be the reason for the variation in results when compared with our findings. In another study by Srinivasan et al, 29 milled (AvaDent) and printed (NextDent) resins exhibited comparable surface roughness, which is in disagreement with the current study. This might be due to the difference in polishing techniques, printing orientation, and layer thickness.

The type of printer, printing technologies, and printing resins were the variables available among the three groups of 3D-printed specimens tested in the current study. The printing technologies of the three printers used were as follows: the NextDent and FormLabs printers were based on SLA, and the ASIGA printer was based on digital light projection (DLP) technology. NextDent specimens exhibited higher surface roughness than those of FormLabs and ASIGA. Since the printing parameters (layer thickness and printing orientation) were similar in all 3D-printed groups, and the printing technology of NextDent and FormLabs was the same (SLA), therefore the variation in surface roughness might be related to other parameters and limitations within each printer or printing material.

The contact angles of milled resins were significantly lower than those of HP and 3D-printed resins in the present study. However, Al-Dwairi et al 15 16 found that HP had a lower contact angle than milled and 3D-printed resins. Differences in results could be related to the variations in the study designs and tested materials. In addition, they compared the properties of milled and 3D-printed resins to HP resins in two separate studies. 15 16 Comparison of contact angle results between our study and previously published research was difficult due to the low number of studies testing contact angles of CAD/CAM materials.

*C. albicans* adhesion in the present study was not significantly affected by the manufacturing technique, as there was no significant difference in *Candida* adherence to the surfaces of the tested materials. However, the lowest count was found with milled CAD/CAM resins followed by printed resin, except NextDent specimens, which showed higher *Candida* count than HP PMMA. A previous study 12 compared *C. albicans* adhesion between different CAD/CAM resins (milled and 3D printed) and HP PMMA after 16 hours of incubation and reported similar results. They demonstrated that the time of incubation affected *Candida* adhesion. Increasing the incubation time resulted in microbial biofilm formation and increased *Candida* adhesion independent of the surface roughness. 12 In the present study, the incubation time was 48 hours. This could explain the results of no difference between the tested groups regarding *C. albicans* adhesion. Previous studies reported lower *Candida* adhesion on milled resin compared with HP resin after short incubation periods (90 minutes and 2 hours) than the one followed in our study. 8 18 Also, the tested materials were different than those tested in the present study. A recent report noted that the printing technology (SLA and DLP) does not influence *C. albicans* adhesion to 3D-printed denture bases, supporting the results in the present study. 30 Shim et al 31 investigated the effect of printing orientation on *Candida* adhesion and found a significantly lower *Candida* count when a 90-degree orientation was used in comparison to 0 and 45 degrees. Accordingly, the vertical printing orientation followed in our study might be the reason for no differences of *C. albicans* adhesion among 3D-printed, HP, and milled resins, even in NextDent specimens, which showed a significantly higher surface roughness.

The results of the present study showed that contact angle was significantly correlated with *C. albicans* adhesion, while surface roughness showed no significant correlation. Milled resins tested in this study had the lowest values of contact angle compared with HP and 3D-printed resins. In addition, they also had the least number of *C. albicans* count but without significant difference compared with printed and HP resins. The correlation between surface hydrophobicity and *Candida* adhesion has been confirmed previously. 4 It was demonstrated that a decrease in contact angle was associated with less *Candida* adhesion. 4 Murat et al 8 reported similar findings of lower contact angle and *C. albicans* adhesion with milled denture base resin than HP. However, contrary to our results, they reported lower surface roughness of milled resin than HP, and significant correlation between the increase in surface roughness and the number of attached *C. albicans* cells, while hydrophobicity (high contact angle) showed no correlation. Variation in results might be related to differences in the tested materials, polishing techniques, and then exposing the specimens to thermal cycling. Moreover, printed resins were not evaluated in their study.

The findings of the current study presented no difference regarding *C. albicans* adhesion to CAD/CAM and HP denture base resins. However, the lowest count was found with milled resins. High contact angle was significantly correlated with higher *C. albicans* adhesion, while surface roughness showed no significant correlation. In between printed resins and compared with milled and HP resins, NextDent showed the highest surface roughness and contact angle. However, other tested 3D-printed resins showed similar properties to HP. Looking at the results of this study, the tested 3D-printed resins could be used clinically for the construction of complete dentures with similar possibility of *C. albicans* adhesion as HP and milled resins. Thus, incidence of denture stomatitis is expected to be similar among denture base materials manufactured by conventional heat polymerization, milling, or 3D-printing technologies. Based on the present results, the tested denture base materials when used clinically for construction of denture bases would show comparable adhesion of *C. albicans* irrespective to their method of fabrication (conventional, milled, and 3D printed). However, the present findings should be interpreted with caution due to its *in vitro* nature. Exposure to artificial saliva with various pH affects the mechanical and surface roughness of conventional and CAD/CAM denture base resins. 32 Therefore, clinical studies are required to support the present findings, after exposing the tested materials to masticatory forces, oral flora, saliva, denture cleansing, and food and beverages with varying temperatures.

The present study included more than one brand from each CAD/CAM manufacturing techniques, in addition to the use of two printing technologies, SLA and DLP, which would add validity to the results reported by each manufacturing technique. The biofilm assay by determination of CFU is a common microbiological research technique; however, this process is laborious, tedious, and time-consuming. 23 Future research should focus on studying the biofilm of *C. albicans* using other methods such as Cell Proliferation Assay Kits. This study is limited as the effect of aging or denture cleaning routines on the tested properties was not tested. Further investigations are required to test the effect of aging and beverage consumption on different characteristics of CAD/CAM resins. The differences between 3D-printed resins tested in the present study require further investigations to understand the factors causing these variations.

## Conclusion

The adhesion of *C. albicans* to the surfaces of milled, 3D-printed, and HP denture base resins was similar; however, the lowest count was found in milled resins. Surface roughness of milled and 3D-printed resins was the same as that of HP, except NextDent, which showed the highest value. Milled resins had significantly lower contact angles compared with other tested groups.
